# Strategies, personnel, and diversity of pediatric antimicrobial stewardship programs in the United States: Strategies and composition of US pediatric ASPs

**DOI:** 10.1017/ash.2025.18

**Published:** 2025-02-25

**Authors:** Christina S. Manice, Alexander S. Plattner, Edan Leshem, Jason G. Newland, Mari M. Nakamura

**Affiliations:** 1 Division of Infectious Diseases, Boston Children’s Hospital, Boston, MA, USA; 2 Antimicrobial Stewardship Program, Boston Children’s Hospital, Boston, MA, USA; 3 Department of Pediatrics, Harvard Medical School, Boston, MA, USA; 4 Division of Infectious Diseases, Department of Pediatrics, Washington University School of Medicine, St. Louis, MO, USA; 5 Institute for Informatics, Data Science, and Biostatistics, Washington University School of Medicine, St. Louis, MO, USA; 6 Division of Infectious Diseases, Nationwide Children’s Hospital and The Ohio State University, Columbus, OH, USA

## Abstract

**Objective:**

We sought to characterize US pediatric antimicrobial stewardship programs (ASPs), including their hospital demographics, staffing, funded full-time equivalents (FTEs) by hospital size, and relative emphasis on recommended stewardship strategies. We examined the self-reported characteristics of ASP personnel with regard to discipline, race, ethnicity, gender identity, and years of experience in antimicrobial stewardship.

**Design:**

Descriptive two-part survey.

**Setting:**

Pediatric ASPs at hospitals participating in Sharing Antimicrobial Reports for Pediatric Stewardship (SHARPS), a pediatric quality improvement collaborative of >70 children’s hospitals.

**Participants:**

Survey distributed to 82 US pediatric ASPs, excluding hospitals without pediatric ASPs. Part I completed by ASP leader (physician or pharmacist). Part II distributed to ASP team members.

**Methods:**

Part I addressed hospital demographics, ASP funding, and program choices related to the CDC’s 2019 Core Elements of Hospital Antibiotic Stewardship Programs. Part II requested that participants anonymously self-identify race, ethnicity, gender identity, training, and duration of ASP experience. Descriptive statistics performed.

**Results:**

Sixty-two ASPs responded: 61 (98%) with formal ASP, 40 (65%) from freestanding children’s hospitals. 40 (65%) co-led by an ASP physician and pharmacist. 60 (97%) reported dedicated inpatient physician FTE, 57 (92%) dedicated inpatient pharmacist FTE. Most programs (35 [58%]) reported inadequate staffing support. The 125 ASP professionals who completed Part II predominantly self-reported as White (89 [71%]), with fewer self-reporting as Asian (9 [15%]) or Black (4 [3%]).

**Conclusion:**

US pediatric ASPs have achieved substantial progress in meeting the CDC Core Elements, but many report insufficient resources. We identified underrepresentation in the ASP workforce.

## Introduction

Antimicrobial stewardship programs (ASPs) encourage judicious, effective, and safe antimicrobial use, thereby preventing emergence of resistance, improving outcomes, and reducing costs.^1–4^ Hospital ASPs are recommended by the Infectious Disease Society of America, Pediatric Infectious Diseases Society, American Academy of Pediatrics, and Centers for Disease Control and Prevention (CDC) and are required by the Centers for Medicare and Medicaid Services and The Joint Commission (TJC).^1,5–8^ A systematic review of 146 studies from 2000 to 2017 found that ASPs were associated with decreased antimicrobial use and resistance, with reduced (or unchanged) lengths of stay, mortality rates, and readmission rates and overall hospital cost savings.^9^ A 2018 survey of US children’s hospitals by McPherson et al. showed that 94% had formal ASPs.^5^

In the current study, we investigated the change in prevalence of US pediatric ASPs since the 2018 McPherson study. We assessed staffing, including evaluating funded full-time equivalents (FTEs) by hospital size, and characterized relative emphasis on recommended stewardship strategies. Understanding the predominant activities and composition of pediatric ASPs will facilitate future analysis of the effectiveness of different ASP strategies and structures.

We also examined the self-reported characteristics of ASP personnel with regard to discipline, race, ethnicity, gender identity, and years of experience working in antimicrobial stewardship. This assessment of diversity and inclusion among programs was a first step to developing interventions to increase these program characteristics and thus enable ASPs to better facilitate more equitable pediatric care, as well as foster inclusive environments in which racism, discrimination, and bias are not tolerated.

## Methods

### Study population

From March through June 2023, we surveyed US pediatric hospitals participating in Sharing Antimicrobial Reports for Pediatric Stewardship (SHARPS), a quality improvement collaborative of >70 children’s hospitals focused on sharing data and benchmarking antimicrobial use to improve prescribing for hospitalized children.^5,10^ We invited 82 ASP leaders (pediatric infectious diseases [ID] physicians or pharmacists) to participate, requesting 1 response per institution. We distributed the survey and managed responses using REDCap (Research Electronic Data Capture), a secure, web-based application designed to support data capture for research studies.^11,12^ Institutions without a dedicated pediatric ASP were excluded. Survey completion indicated consent to participate.

The study was concluded to be an exempt study under US federal regulations [45 Code of Federal Regulations 46.104(d)(4)] by the Washington University School of Medicine and Boston Children’s Hospital Institutional Review Boards. The Boston Children’s Hospital Office of Health Equity and Inclusion also reviewed the survey.

### Survey development

The survey was developed by the authors, who comprised pediatric ID physicians with direct experience in leading or participating in ASPs and an undergraduate working in pediatric ID research. Questions were derived in part from insights from focus groups conducted with pediatric ASP physicians and pharmacists by two of the authors (CSM and MMN) in 2022.^13^ Questions were further generated based on earlier surveys regarding pediatric ASPs and on CDC and TJC recommendations for ASPs.^4,6,7,10^ To establish content validity, investigators reviewed and revised each question to ensure clarity, significance, and applicability. A pilot survey was evaluated by non-investigator ASP experts to confirm relevance, readability, and reasonable length.

### Survey content and data analysis

The survey consisted of 2 parts. Part I contained questions regarding hospital demographics (e.g., name, location, hospital type [freestanding children’s hospital versus pediatric hospital within adult center], and hospital size [number of beds]) and ASP staffing and funding (FTEs by care setting [inpatient or outpatient] and role [e.g., physician, pharmacist]). We also asked about each program’s use of strategies recommended in the CDC’s 2019 Core Elements of Hospital Stewardship Programs,^6^ including their relative emphasis on prospective audit and feedback (PAF) versus preauthorization and use of these strategies for different antimicrobials/antimicrobial classes. ASP leaders completed Part I, then distributed a link for Part II of the survey to their team members. Part II, which was completely anonymous and voluntary, asked respondents to self-identify their race, ethnicity, and gender identity and to report their training and duration of work in antimicrobial stewardship.

We conducted descriptive analyses of survey responses, including median funded FTEs by role, frequencies of ASP team member characteristics by role, and frequencies of PAF or preauthorization use overall and by antimicrobial/antimicrobial class. To characterize the geographic distribution of participating ASPs, we assigned them to the 9 regions used in the American Hospital Association’s (AHA) Annual Survey.^14^ We assessed whether FTEs/hospital bed differed based on whether programs reported adequate staffing using the Wilcoxon rank-sum test, defining a 2-sided *P* < .05 as statistically significant. We used Stata 17 (StataCorp). In addition, we evaluated the distribution of FTEs by hospital size and type using R v4.3.1 and generated graphs using ggplot2 package v3.4.4 (R Foundation for Statistical Computing).

## Results

### ASP program characteristics

**Figure 1.**
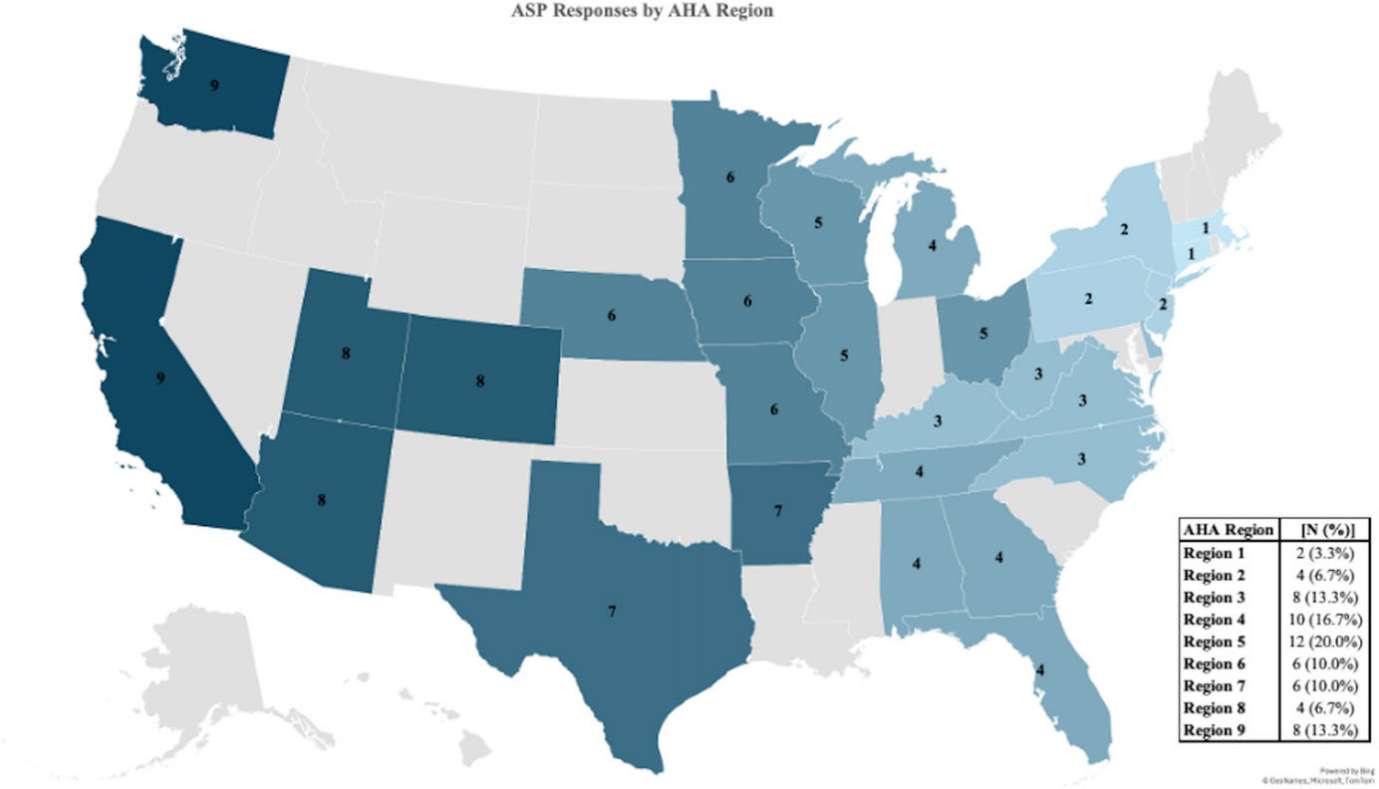
**Survey responses per American Hospital Association Region.** States with programs that responded to the survey are colored and labeled with their corresponding AHA region. Table to right of map reports number of responses per AHA region. ASP, antimicrobial stewardship program; AHA, American Hospital Association.

Sixty-two ASP leaders (44 physicians [71%] and 18 pharmacists [29%]) completed Part I of the survey (Table 1); 2 participants did not complete the section on the CDC’s Core Elements but responded to the other questions. Participating hospitals were geographically well distributed, with responses from each AHA region (Fig. 1). All but 1 of the 62 programs (98%) indicated that they had a formal ASP, defined in the survey as “a comprehensive program that functions continuously to monitor antimicrobial use, that includes fulltime equivalents (FTEs) dedicated for a clinical pharmacist and/or pediatric infectious disease specialist.” Moreover, 53 (88%) programs identified themselves as multidisciplinary (i.e., comprised of pharmacists, physicians, and in some cases, members from other disciplines), and 40 (65%) as co-led by an ASP physician and pharmacist. Most participating institutions were freestanding children’s hospitals (40 [65%]). The median program age was 10 years (IQR: 5–16).

**Table 1.** Antimicrobial stewardship program characteristics

N = 62 programs	
**Hospital Type [n (%)]**	
Freestanding children’s hospital	40 (65%)
Children’s hospital within adult center	21 (34%)
Specialist children’s hospital	1 (2%)
**Hospital Size [median beds (IQR)]**	257 (172–386)
**Median Program Age [median (IQR)]**	10 (5–16)
**Formal ASP**	61 (98%)
**Multidisciplinary^a^ASP**	53 (88%)
**ASP Leadership^b^[n (%)]**	
Physician	8 (13%)
Pharmacist	4 (7%)
Both physician and pharmacist	48 (80%)

a Multidisciplinary defined as combination of pharmacists, physicians, and in some cases, members from other disciplines.

b Two programs did not respond to the question on ASP leadership type.

ASP, antimicrobial stewardship program; IQR, interquartile range.

### ASP staffing and other support

Among ASPs, 60 (97%) reported having dedicated inpatient physician FTEs, while 57 (92%) had dedicated inpatient pharmacist FTEs. In contrast, although 31 (50%) programs engaged in outpatient stewardship, only 11 (36%) had FTEs for any role in outpatient stewardship, with 9 (15%) having physician FTEs and 5 (8%) having pharmacist FTEs (Table 2). Programs reported higher median inpatient FTEs for pharmacists (1 [IQR: 0.5–1]) compared with physicians (0.36 [IQR: 0.26–0.5]). Over half of programs (35 [58%]) reported inadequate financial resources for staffing from hospital leadership. Thirty (50%) programs indicated inadequate financial resources for information technology (IT). The 1 program that reported not meeting criteria for a formal pediatric ASP cited as reasons lack of funding and lack of support from hospital administration.

**Table 2.** Antimicrobial stewardship program staffing support

N = 62 programs	
**Positions with Dedicated Inpatient FTE (N = 62)**	
Physician	60 (97%)
Pharmacist	57 (92%)
Nurse	0
Nurse practitioner	1 (2%)
Data analyst	29 (47%)
Infection preventionist	3 (5%)
Other^a^	2 (3%)
**Median Inpatient FTEs by Role (IQR)**	
Physician	0.36 (0.26–0.50)
Pharmacist	1.0 (0.5–1.0)
Data analyst	0.2 (0.2–0.3)
**Number of Physicians with Inpatient FTEs**	
1	43 (72%)
2	14 (23%)
3	3 (5%)
**Number of Pharmacist with Inpatient FTEs**	
1	37 (65%)
2	14 (25%)
3	6 (11%)
**Number of Data Analysts with Inpatient FTEs**	
0	2 (7%)
1	24 (83%)
2	3 (10%)
**Number (%) of Programs Engaging in Outpatient ASP Activities (With or Without Dedicated FTEs)**	31 (50%)
With dedicated FTEs for outpatient ASP, any role	11 (36)
With dedicated FTEs for outpatient physician	9 (15%)
With dedicated FTEs for outpatient pharmacist	5 (8%)

ASP, antimicrobial stewardship program; FTE, full-time equivalent; IQR, interquartile range.

a Other refers to physician assistant or program manager.

**Figure 2.**
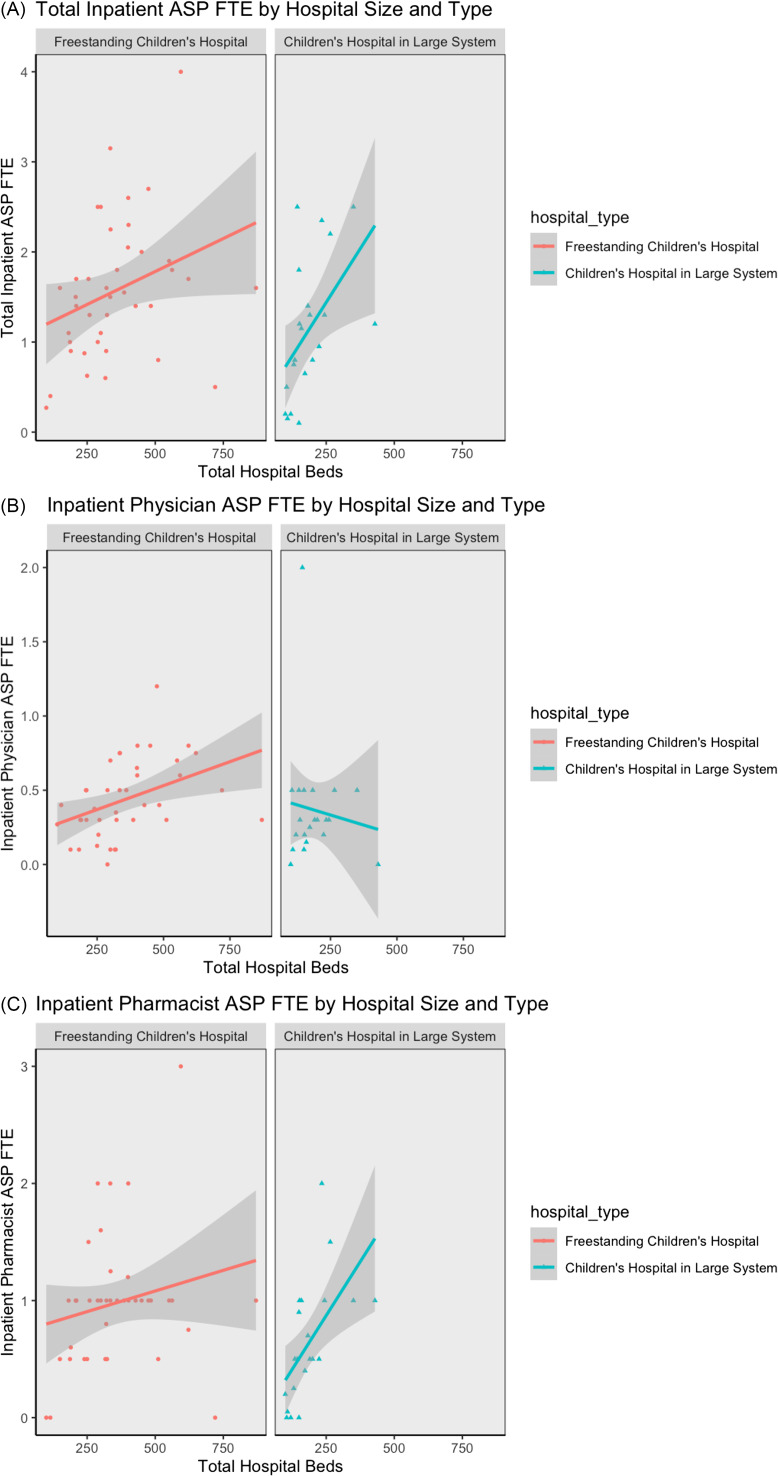
**Inpatient dedicated full-time equivalents (FTE) for antimicrobial stewardship program staff by hospital size (number of beds), stratified by hospital type.** (A) Total inpatient FTE. (B) Inpatient physician FTE. (C) Inpatient pharmacist FTE. ASP, antimicrobial stewardship program; FTE, full-time equivalent.

As hospital size increased, total inpatient FTEs increased for both freestanding children’s hospitals and children’s hospitals within adult centers (Fig. 2, Panel A). Inpatient physician FTEs increased with hospital size for freestanding children’s hospitals but decreased for children’s hospitals within adult centers (Fig. 2, Panel B); the latter finding was driven by one outlier as excluding that program’s response resulted in an upward trend. In contrast, inpatient pharmacist FTEs rose with bed size for both hospital types, although the increase was more pronounced for children’s hospitals within adult centers than freestanding children’s hospitals (Fig. 2, Panel C). The median FTEs/hospital bed was significantly higher for programs with perceived adequate staffing support than for those without (0.0074 [IQR: 0.0036–0.0083] versus 0.0047 [IQR: 0.0028–0.0065] FTEs/bed), *z* = −1.972, *p* = .0486.

### ASP workforce demographics and characteristics

Part II of the survey was completed by 125 ASP professionals comprising mostly physicians (51 [48%]) and pharmacists (65 [52%]) (Table 3). The most frequently self-reported race was White (89 [71%]), with fewer reporting their race as Asian (9 [15%]), Black (4 [3%]), or other (6 [5%]) or preferring not to answer (8 [6%]). Most participants identified as women (84 [67%]) or men (36 [29%]); 5 (4%) preferred not to answer. No other gender identity was self-reported. The median time working in ASP was 6 years (IQR: 3–10). Of the pharmacist respondents, 33 (51%) reported completing formal ASP training, with 26 (40%) having trained in pediatric ID, 22 (34%) in adult ID, and 31 (48%) in pediatrics but not ID.

**Table 3.** Antimicrobial stewardship program workforce and demographics by self report

N = 125 ASP professionals							
Employee Type N (%)	Physicians	Pharmacists	RN	Data Analyst	IP	Other	Overall
**Overall**	51 (41%)	65 (52%)	1 (1%)	6 (5%)	1 (1%)	1 (1%)	125
**Race^a^**							
Asian	9 (18%)	7 (11%)	0 (0%)	3 (50%)	0 (0%)	0 (0%)	19 (15%)
Black or African-American	2 (4%)	2 (3%)	0 (0%)	0 (0%)	0 (0%)	0 (0%)	4 (3%)
White	34 (67%)	50 (77%)	1 (100%)	3 (50%)	1 (100%)	0 (0%)	89 (71%)
Other	5 (10%)	1 (2%)	0 (0%)	0 (0%)	0 (0%)	0 (0%)	6 (5%)
Prefer not to answer	2 (4%)	5 (8%)	0 (0%)	0 (0%)	0 (0%)	1 (100%)	8 (6%)
**Gender^b^**							
Woman	32 (63%)	45 (69%)	1 (100%)	5 (83%)	1 (100%)	0 (0%)	84 (67%)
Man	17 (33%)	17 (26%)	0 (0%)	1 (17%)	0 (0%)	1 (100%)	36 (29%)
Prefer not to answer	2 (4%)	3 (5%)	0 (0%)	0 (0%)	0 (0%)	0 (0%)	5 (4%)

ASP, antimicrobial stewardship program, RN, registered nurse; IP, infection preventionist.

a None of the participants self-identified as American Indian/Alaska Native or Native Hawaiian/Other Pacific Islander.

b None of the participants self-identified as non-binary, transgender man/female-to-male (FTM), transgender woman/male-to-female (MTF), gender non-binary/genderqueer/gender nonconforming, agender, or bigender.

### ASP strategies

#### Preauthorization

**Figure 3.**
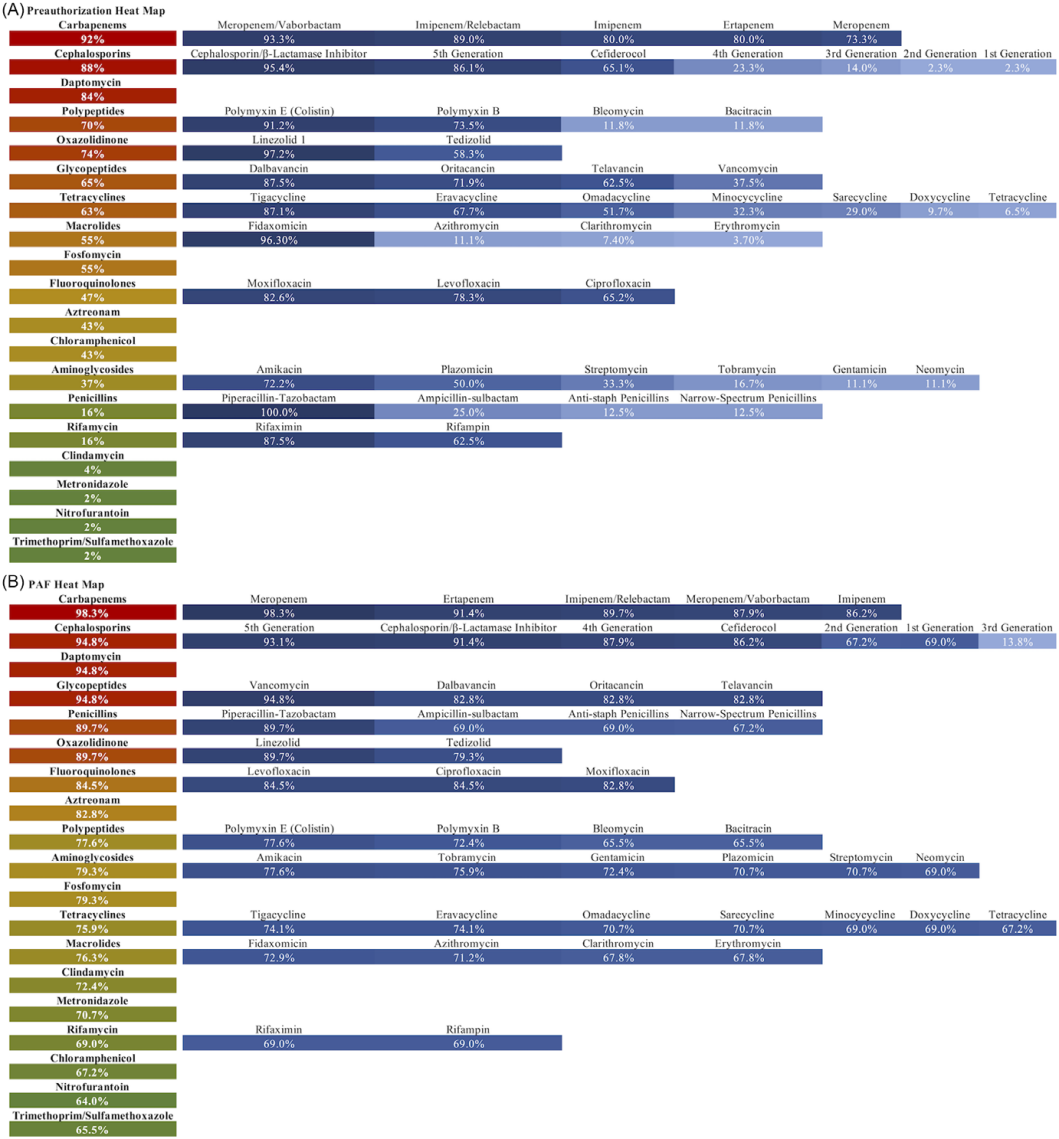
**Frequency of use of preauthorization (Panel A) or prospective audit and feedback (PAF) by antibiotic class/antibiotic.** The percentage displayed for each antibiotic class is the percentage of programs that required preauthorization or performed audit and feedback for any drug within that class. Red denotes increased frequency of use of the strategy, whereas green denotes decreased frequency of use. For individual antibiotics, darker blue denotes increased frequency of use of the strategy, whereas lighter blue denotes decreased frequency of use.

Among all ASPs, 49 (79%) performed at least some preauthorization. Carbapenems were the most restricted class (92% of programs that used preauthorization). Cephalosporins were restricted by 88% of programs, among whom 95% required preauthorization for novel cephalosporin/beta-lactamase inhibitor agents versus only 23% for cefepime (Fig. 3, Panel A). Six programs (14%) required formal ID consultation for every approval, while 19 programs (43%) required formal ID consultation for some approvals. Over half of programs (30 [61%]) allowed initiation of restricted antimicrobials overnight but required authorization for ongoing use.

#### Prospective audit and feedback

Among all ASPs, 58 (94%) reported some use of PAF. Carbapenems were reviewed by 98% of programs that used PAF. Cephalosporins were reviewed by 95% of programs, with 93% of those auditing ceftaroline, 91% novel cephalosporin/beta-lactamase inhibitor agents, and 88% cefepime (Fig. 3, Panel B). Of ASPs performing PAF, 37 (64%) reported using handshake stewardship. Thirty-one programs (50%) tracked and documented PAF recommendations in the electronic health record (EHR), whereas 19 (31%) tracked recommendations outside the EHR.

#### Relative emphasis on preauthorization versus prospective audit and feedback

When asked about their predominant strategy, 3 programs (5%) indicated preauthorization, 32 (53%) PAF, and 25 (42%) a hybrid of preauthorization and PAF. Among preauthorization programs, 100% (n = 3) restricted carbapenems and oxazolidinones, and 67% (n = 2) restricted cephalosporins and daptomycin (Table 4). Among the 32 PAF programs, the percentage that used preauthorization versus PAF were 86% versus 100% for carbapenems, 62% versus 100% for oxazolidinones, and 76% versus 100% for daptomycin. The percentage of PAF programs that used preauthorization versus PAF for cephalosporins was the same at 91%. Finally, among hybrid programs, the percentage that used preauthorization versus PAF was the same for carbapenems at 96% and for cephalosporins at 88%. The percentage of hybrid programs that used preauthorization versus PAF was 80% versus 88% for oxazolidinones, and 92% versus 88% for daptomycin (Table 4).

**Table 4.** Frequency of preauthorization or prospective audit and feedback by antibiotic class/antibiotic, stratified by predominant antimicrobial stewardship program strategy

	Predominant ASP Strategy		
	Preauthorization N = 3	Prospective audit and feedback (PAF) N = 21	Hybrid N = 25
	% (n)	% (n)	% (n)
**Carbapenems**			
Preauthorization	100% (3)	86% (18)	96% (24)
PAF	100% (2)	100% (21)	96% (23)
**Cephalosporins**			
Preauthorization	67% (2)	91% (19)	88% (22)
PAF	100% (2)	91% (19)	88% (21)
**Ceftriaxone**			
Preauthorization	33% (1)	10% (2)	12% (3)
PAF	0% (0)	19% (4)	8% (2)
**Cefazolin**			
Preauthorization	0% (0)	5% (1)	0% (0)
PAF	100% (2)	91% (19)	63% (15)
**Daptomycin**			
Preauthorization	67% (2)	76% (16)	92% (23)
PAF	100% (2)	100% (21)	88% (21)
**Oxazolidinones**			
Preauthorization	100% (3)	62% (13)	80% (20)
PAF	100% (2)	100% (21)	88% (21)

Percentage (n) of programs who reported performing preauthorization or prospective audit and feedback (PAF) by antibiotic class/antibiotic, stratified by their self-identified predominant antimicrobial stewardship strategy. Only programs that reported performing any frequency of preauthorization or PAF were included in analyses of each strategy: For example, programs that reported performing no PAF at all (n = 2) were not included in assessments of PAF.

#### Care setting of ASP activities

Among all ASPs, 60 (97%) performed ASP activities in inpatient settings, 45 (73%) in the Emergency Department, 29 (47%) at outpatient facilities within the hospital, and 3 (5%) in other settings. For programs performing PAF, 54 (93%) performed audits in all hospital units.

#### Other ASP activities

Nearly all programs (95% [n = 59]) had developed ≥2 evidence-based guidelines on antibiotic use. The most commonly addressed conditions were community-acquired pneumonia (90%), urinary tract infections (82%), and surgical prophylactic antibiotics (82%). However, only 73% (n = 44) of programs measured adherence to ≥1 guideline. Finally, 100% (n = 60) of programs developed an annual antibiogram, which was pediatric-specific for all but 1 program (98%).

### Tracking, monitoring, and sharing

Among all ASPs, antibiotic use reports were monitored regularly by 97% (n = 58), of whom 98% (n = 57) monitored days of therapy per 1,000 patient days. Regarding National Healthcare Safety Network (NHSN) reporting, 68% (n = 42) reported antibiotic use data and 24% (n = 15) reported antibiotic resistance data, leaving 23% (n = 14) who did not report to NSHN. Programs also shared data with the ASP Committee (87%), hospital administrators (84%), Pharmacy and Therapeutics Committee (76%), front-line clinicians (66%), and other stakeholders (21%; e.g., ID division, Infection Prevention and Control Program, Microbiology Laboratory, and state health department).

## Discussion

Our study provides a current understanding of the prevalence, staffing, resources, composition, and activities of US pediatric ASPs. While the percentage of ASPs reporting formal programs markedly increased from 38% to 94% from 2014 to 2018, our study found a further increase to 98%.^5,15^ TJC began requiring hospital ASPs in 2017, perhaps helping to drive this change and reflecting the potential positive impact of evidence-based regulatory requirements.^16^

We found that 88% of programs were multidisciplinary, with 80% co-led by pharmacists and physicians—a leadership structure highlighted in the 2019 update of the CDC Core Elements as particularly effective (Table 1).^6^ A mixed-methods study determined that leadership by ID pharmacists and physicians with higher dedicated effort for stewardship was among the factors associated with stewardship excellence.^17,18^ At the same time, a white paper on the knowledge and skills required for ASP leaders noted that while deep ID expertise is valuable, skills and knowledge in other domains such as microbiology, infection control, measurement and analysis, and IT are also key, underscoring the need for multidisciplinary teams.^17^

Our study revealed similar dedicated FTEs for expert personnel as McPherson et al (2018).^5^ The median physician FTEs was slightly higher in our study at 0.36 (IQR: 0.26–0.5), compared with 0.3 (IQR: 0.2–0.5), while the median pharmacist FTEs was unchanged at 1.0 FTE (IQR: 0.5–1.0).^5^ Our results showed 47% of programs had a data analyst, an increase from 35% in 2018,^5^ with a current reported median data analyst FTE of 0.2 (IQR: 0.2–0.3); 24 programs had 1 funded analyst, while 3 programs had 2 analysts (Table 2). Examining the relationship between staffing support and hospital size, we determined that overall and pharmacist FTEs increased with number of beds for all hospital types. Physician FTE actually decreased with number of beds for pediatric hospitals within larger hospital systems (Fig. 2, Panel B), but this could be due to 1 outlier, as the trend increased with that program’s response removed.

Even with improvements in staffing support, over half of programs (35 [58%]) still reported insufficient resources for staffing from hospital leadership. Optimal ASP staffing ratios have not yet been defined. While the 2023 TJC requirements state, “The hospital allocates financial resources for staffing and information technology to support the antibiotic stewardship program,” neither TJC requirements nor the CDC Core Elements specify a target FTE/bed ratio or specific FTE standards per stewardship role.^6,7^ The median FTEs/bed for programs reporting adequate staffing support was 0.0074 (e.g., 2.22 FTEs for a 300-bed hospital), suggesting a rough target. However, this crude calculation fails to capture nuances such as types of FTE (e.g., pharmacist, analyst) and hospital case-mix (e.g., acuity, complexity). Examining how ASP effectiveness, as measured by performance on measures such as case-mix-adjusted antibiotic use, correlates with staffing ratios would be a valuable question for further study.

Both TJC and the CDC emphasize preauthorization and/or PAF as key antimicrobial stewardship interventions.^6,7^ While all participants in our study reported using 1 or both of these strategies, programs varied in their relative emphasis, with a small fraction focusing on preauthorization, about half focusing on PAF, and approximately 40% using a hybrid. This finding was consistent with those from our prior focus group study with a smaller sample of ASPs.^13^ Programs also varied in relative emphasis on preauthorization versus PAF for specific antimicrobial classes or antimicrobials, with preauthorization used somewhat less frequently than PAF for many antimicrobial classes/antimicrobials (Table 4, Fig. 3).

Most programs (95% [n = 59]) met TJC’s requirement of using ≥2 evidence-based guidelines for common infections, but fewer (73% [n = 44]) reported tracking guideline adherence. This gap may reflect challenges of such measures, which entail not just evaluating antibiotic use, but also assessing appropriateness of use—a more difficult task to accomplish with automated methods. Increased support for IT and data analysis, collaboration with hospital quality improvement programs, and innovative measurement techniques such as use of natural language processing to facilitate chart review could aid in these efforts.^19^

Our study was the first to examine the self-reported composition of pediatric ASPs with regard to race, ethnicity, and gender identity. We found low racial diversity and low inclusion of historically underrepresented groups among ASPs, with a majority of respondents (71%) self-reporting their race as White, fewer as Asian (15%), and only 3% as Black (Table 3). This finding may reflect underrepresentation more generally among pediatricians: According to 2023 US Census data, 13.7% of the population identifies as Black or African American, whereas 2017 American Association of Medical Colleges (AAMC) data demonstrated that only 7.4% of pediatricians identified as Black or African American.^20,21^ With regard to gender identity, 67% of respondents reported female gender, with 63% of physicians reporting female gender, similar to the AAMC statistic that women comprised 72.3% of pediatrics residents and 63.3% of physicians in practice.^22–24^ We believe that ASPs, like hospitals, as urged by the American Academy of Pediatrics, should advocate for programs that diversify the pediatric workforce as a way to reduce implicit bias and improve the safety and quality of pediatric care.^25^ A key strategy is improving recruitment, development, and retention of a diverse ASP workforce, which will in turn help ASPs to eliminate structural racism from antimicrobial stewardship guidelines and practices and to lead in development and tracking of metrics for diversity, equity, and inclusion in antimicrobial stewardship.

Our study had multiple strengths, including representation from 62 geographically diverse pediatric ASPs out of the 83 programs invited to participate. Survey responses provided comprehensive information regarding staffing, funding, and activities performed by ASPs. Moreover, our survey was the first to investigate the composition of ASPs and identify a lack of racial representation within the ASP workforce so that it can be further studied and addressed.

Our study also had limitations. Given the survey’s descriptive nature and the relatively small number of US children’s hospitals and thus pediatric ASPs, we lacked power for quantitative comparisons. There is potential for bias as ASPs that participated in the survey may differ from non-participants. For example, programs with inadequate staffing or other support might have been more compelled to share this issue by completing the survey than adequately resourced programs. Finally, while Part II of the study was anonymous, given the potentially sensitive nature of the questions, it is possible that some ASP professionals did not feel comfortable representing themselves candidly and either chose “prefer not to answer” responses or did not complete the survey at all.

In conclusion, we found that US pediatric ASPs have made great progress toward meeting the 2019 CDC Core Elements and the TJC’s 2023 requirements for hospital antimicrobial stewardship, though most still report insufficient resources. We identified underrepresentation in the ASP work force and hope that future studies can explore improving representation and equitable pediatric care within antimicrobial stewardship. We hope that understanding the current state of pediatric ASPs will spur studies on how stewardship funding and activities (e.g., preauthorization versus PAF) relate to outcomes, thus illuminating the comparative effectiveness of different ASP structures and strategies.

## Supporting information

Manice et al. supplementary material Manice et al. supplementary material

## Supplementary material

For supplementary material accompanying this paper visit http://doi.org/10.1017/ash.2025.18. click here to view supplementary material
