# Supplementary material for: Strategies, personnel, and diversity of pediatric antimicrobial stewardship programs in the United States: Strategies and composition of US pediatric ASPs
Source: Antimicrob Steward Healthc Epidemiol. 2025 Feb 25;5(1):e59. doi: 10.1017/ash.2025.18 (PMC11869051; doi:10.1017/ash.2025.18)
Supplement: Manice et al. supplementary material [file S2732494X2500018Xsup001.pdf]

SUPPLEMENT

Strategies, staffing, and diversity of pediatric antimicrobial stewardship programs in the United States

Christina S. Manice, MD,[1,2,3] Alexander S. Plattner, MD, MBA,[4,5] Edan Leshem,[4] Jason G. Newland, MD, MEd,[6] Mari M. Nakamura, MD, MPH [1,2,3]

Affiliations:

[1]Division of Infectious Diseases, Boston Children's Hospital, Boston, MA, USA

[2]Antimicrobial Stewardship Program, Boston Children's Hospital, Boston, MA, USA

[3]Department of Pediatrics, Harvard Medical School, Boston, MA, USA

[4]Division of Infectious Diseases, Department of Pediatrics, Washington University School of Medicine, St. Louis, MO, USA

[5]Institute for Informatics, Data Science, and Biostatistics, Washington University School of Medicine, St. Louis, MO, USA

[6]Division of Infectious Diseases, Nationwide Children's Hospital and The Ohio State University, Columbus, OH, USA

Table of Contents

Survey, Part I 2

Survey, Part II 14

Survey, Part I

ASP Survey Start

Please complete the survey below to the best of your knowledges. Feel free to consult others, as needed.

If you have any questions, please contact Alex Plattner (aplattner@wustl.edu).

Thank you!

Full Name: __________________________________

Email: __________________________________

Hospital Demographics

Hospital name: __________________________________

Hospital location (city, state): __________________________________

What is your position within the hospital?

- ◯ Physician
- ◯ Pharmacist
- ◯ Nurse Practitioner
- ◯ Registered Nurse
- ◯ Data Analyst
- ◯ Infection Prevention Specialist
- ◯ Other ______

Select the option that best describes your hospital.

- ◯ Freestanding children's hospital (with or without affiliated pediatric clinics)
- ◯ Children's hospital contained within a larger adult/pediatric healthcare system
- ◯ Specialist Children's Hospital (e.g. Children's Cancer Center)
- ◯ Pediatric/Neonatal Units within a Specialist Adult Hospital (e.g. cancer hospital) with a pediatric ward
- ◯ Other ______

Total licensed hospital bed count (only pediatric beds if in a hospital that has adult beds): __________________________________

For the purposes of this survey we define an Antimicrobial Stewardship Program (ASP) as follows:
ASP a comprehensive program that functions continuously to monitor antimicrobial use, that includes fulltime equivalents (FTEs) dedicated for a clinical pharmacist and/or pediatric infectious disease specialist.

According to the definition above, does your hospital have a pediatric ASP?

- ◯ Yes
- ◯ No

You stated that your hospital does not currently have a pediatric ASP. Which of the following reasons best describe why? (Select all that apply.)

- ☐ Lack of support from hospital administration
- ☐ Lack of time
- ☐ Lack of personnel
- ☐ Lack of funding
- ☐ Program is currently in the development stage
- ☐ Other ______

projectredcap.org REDCap®

What year was your formal pediatric ASP established?

____

Was there informal ASP activity prior?

◯ Yes
◯ No

When did this informal activity start?

____

Provider FTE

Please complete the survey below.

Thank you!

The following questions refer to inpatient ASP settings.

Indicate whether your hospital has the following positions with dedicated FTE to inpatient pediatric ASP (select all that apply):

______

Total Physicians: ______ Total Physician FTE: ______
Total Pharmacists: ______ Total Pharmacist FTE: ______
Total Registered Nurses: ______ Total Registered Nurse FTE: ______
Total Nurse Practitioners: ______ Total Nurse Practitioner FTE: ______
Total Data Analysts: ______ Total Data Analyst FTE: ______
Total Infection Preventionists: ______ Total Infection Preventionist FTE: ______
Total [inpatient_other]s: ______ Total [inpatient_other] FTE: ______

How many of your ASP physicians are Pediatric ID trained? __________________________________

You indicated that [pedidtraining_physician] ASP physician(s) are Pediatric ID trained. What are other ASP physician training types? __________________________________

The following questions are calculated based on your inpatient ASP.

How many total providers receive FTE for your inpatient ASP? __________________________________

How much total FTE is dedicated to your inpatient ASP? __________________________________

The following questions refer to outpatient ASP settings.

Does your ASP perform outpatient stewardship?
◯ Yes
◯ No

Does your ASP have dedicated FTE for outpatient stewardship works?
◯ Yes
◯ No

If your ASP does not have dedicated outpatient FTE, ignore this table.

If you answered "Yes" above for outpatient FTE, indicate whether your hospital has the following positions with dedicated FTE for outpatient ASP settings (select all that apply):

______

Total Physicians: ______ Total Physician FTE: ______
Total Pharmacists: ______ Total Pharmacist FTE: ______
Total Registered Nurse: ______ Total Registered Nurse FTE: ______
Total Nurse Practitioners: ______ Total Nurse Practitioner FTE: ______
Total Data Analysts: ______ Total Data Analyst FTE: ______
Total Infection Preventionists: ______ Total Infection Preventionist FTE: ______
Total [outpt_other]s: ______ Total [outpt_other] FTE: ______

How many physicians are Pediatric ID trained?	______________________________
You indicated that [pedidtraining_physician] physician(s) is Pediatric ID trained. What are other physician training types?	______________________________

The following questions are calculated based on your outpatient ASP.

How many total providers receive FTE for your outpatient ASP?	______________________________
How much total FTE is dedicated to your outpatient ASP?	______________________________

CDC 2019 Core Elements

Please complete the survey below.

Thank you!

Hospital Leadership Commitment

To what part of hospital leadership does your ASP report stewardship activities and/or outcomes? (Select all that apply.)

- ☐ Hospital Quality and Safety Leadership
- ☐ Pharmacy Leadership
- ☐ Infection Prevention and Control
- ☐ Division of Pediatric Infectious Diseases
- ☐ Other ______
- ☐ None

How often do you report to hospital quality and safety leadership?

◯ Weekly ◯ Monthly ◯ Quarterly ◯ Semi-Annually ◯ Annually ◯ Other ______

How often do you report to pharmacy leadership?

◯ Weekly ◯ Monthly ◯ Quarterly ◯ Semi-Annually ◯ Annually ◯ Other ______

How often do you report to infection prevention and control leadership?

◯ Weekly ◯ Monthly ◯ Quarterly ◯ Semi-Annually ◯ Annually ◯ Other ______

How often do you report to division of pediatric infectious diseases leadership?

◯ Weekly ◯ Monthly ◯ Quarterly ◯ Semi-Annually ◯ Annually ◯ Other ______

How often do you report to [leadership_other]?

◯ Weekly ◯ Monthly ◯ Quarterly ◯ Semi-Annually ◯ Annually ◯ Other ______

Does your ASP receive adequate financial resources for staffing from hospital leadership?

◯ Yes
◯ No

What additional staffing do you need?

____

Does your ASP receive adequate financial resources for information technology from hospital leadership?

◯ Yes
◯ No

What additional information technology resources do you need?

____

Accountability

Who leads or co-leads your ASP? (Select all that apply.)	☐ ID Physician<br>☐ Other Physician ______<br>☐ Pharmacist<br>☐ Registered Nurse<br>☐ Nurse Practitioner<br>☐ Data Analyst<br>☐ Infection Prevention<br>☐ Other Non-Physician ______
Does your hospital have a multidisciplinary ASP committee?<br><br>A multidisciplinary committee is defined by being composed of a combination of medical staff from different specialties, pharmacy services, nursing staff, infection prevention and control programs, data technicians/analysts, and/or quality improvement experts.	◯ Yes<br>◯ No
How frequently does the committee meet?	◯ Weekly<br>◯ Monthly<br>◯ Quarterly<br>◯ Semi-Annually<br>◯ Annually<br>◯ Other ______

Pharmacy Expertise

What is the training of the pharmacist(s) involved with ASP efforts? (Select all that apply.)	☐ Pediatrics (non-ID)<br>☐ Adult ID<br>☐ Pediatric ID<br>☐ Other ______
Has the pharmacist(s) involved with ASP efforts received any formal training in ASP (e.g., participation in a certificate program)?	◯ Yes<br>◯ No
Does the pharmacist(s) lead or co-lead antimicrobial stewardship implementation efforts?	◯ Yes<br>◯ No

Action

In which of the following hospital departments and services does your ASP perform stewardship activities? (Select all that apply.)	☐ In-patient, hospitalizations<br>☐ Emergency department<br>☐ Out-patient facilities within the hospital<br>☐ Other ______
Does your hospital ASP require pre-authorization of any antimicrobials?	◯ All<br>◯ Some<br>◯ None

For antimicrobials on your formulary, which antimicrobials do you restrict? If an antimicrobial is not on your formulary, leave that option blank.

(Choosing "some" will expand that section for individual drugs in each class - please select all that you restrict from your formulary).

Antibiotics

Penicillins ______

______

Cephalosporins ______

______

Carbapenems ______

______

Aztreonam ______

Aminoglycosides ______

______

Macrolides and/or Derivatives ______

______

Glycopeptides ______

______

Polypeptides ______

______

Fluoroquinolones ______

______

Tetracyclines ______

______

Oxazolidinones ______

______

Rifamycins ______

______

Clindamycin ______

Chloramphenicol ______

Metronidazole ______

Nitrofurantoin ______

Trimethoprim/Sulfamethoxazole ______

Daptomycin ______

Fosfomycin ______
Antivirals ______

______

Antifungals ______
______
Antiparasitics ______
______

Who approves the antimicrobial? (Select all that apply.)	☐ ASP<br>☐ ID fellow or advanced practitioner (outside ASP)<br>☐ ID attending (outside ASP)
Do restricted antimicrobials require a formal ID consult for approval?	◯ Yes, All<br>◯ Yes, Some<br>◯ No
How is approval communicated? (Select all that apply.)	☐ From approver to ordering provider<br>☐ From approver to pharmacy<br>☐ Other ______
Does your ASP require approval to dispense overnight doses of restricted antimicrobials?	◯ Yes<br>◯ No
(Optional) Please include any other details that you feel are important to how your ASP handles restricted antimicrobials:	____
Does your hospital ASP perform prospective audit and feedback of any antimicrobials?	◯ All<br>◯ Some<br>◯ None

For antimicrobials on your formulary, which antimicrobials do you perform audit and feedback?

(Choosing "some" will expand that section for individual drugs in each class - please select all that you audit and feedback from your formulary).

Antibiotics
Penicillins ______

______

Cephalosporins ______

______

Carbapenems ______

______

Aztreonam ______
Aminoglycosides ______

______

Macrolides and/or Derivatives ______

______

Glycopeptides ______

______

Polypeptides ______

______

Fluoroquinolones ______

______

Tetracyclines ______

______

Oxazolidinones ______

______

Rifamycins ______

______

Clindamycin ______
Chloramphenicol ______
Metronidazole ______
Nitrofurantoin ______
Trimethoprim/Sulfamethoxazole ______
Daptomycin ______
Fosfomycin ______
Antivirals ______

______

Antifungals ______
______
Antiparasitics ______
______

Does your ASP utilize handshake stewardship?	◯ Yes<br>◯ No
How does your ASP perform audit and feedback?<br>(eg: Who does audit? Who does Feedback?, Do you review data besides antibiotic orders? How are recommendations/feedback delivered?)	________________________________
Do you audit antimicrobials in all units?	◯ Yes<br>◯ No

Which units do you audit? (Select all that apply.)	☐ PICU (including all non-neonatal ICUs)<br>☐ NICU<br>☐ Oncology/BMT<br>☐ ER<br>☐ General Pediatrics<br>☐ Surgery<br>☐ Other ______
Does your ASP track and document recommendations to clinicians for audit and feedback? (Select all that apply)	☐ Tracked within the EHR<br>☐ Tracked outside the EHR<br>☐ Recommendations not tracked<br>☐ Documented within the EHR, visible to clinicians<br>☐ Documented outside the EHR, visible to clinicians<br>☐ Documentation not visible to clinicians<br>☐ Recommendations not documented
(Optional) Please include any other details that you feel are important to how your ASP performs audit and feedback:	________________________________
What does your ASP emphasize most?	◯ Pre-authorization<br>◯ Prospective audit and feedback<br>◯ Combination of pre-authorization and prospective audit and feedback

Tracking

Does your hospital ASP monitor antibiotic use reports on a regular basis (e.g., monthly, quarterly, etc.)?	◯ Yes<br>◯ No
Do you monitor days of therapy per 1,000 patient days?	◯ Yes<br>◯ No
What other denominator do you use for reporting antibiotic use?	__________________________
Do you report data to the National Healthcare Safety Network (NHSN) Antimicrobial Use Option? (Select all that apply.)	☐ Yes, antibiotic use data<br>☐ Yes, antibiotic resistance data<br>☐ No
Do you use NHSN data for any ASP efforts?	◯ Yes<br>◯ No
Has your hospital ASP implemented evidence-based guidelines for prescribing antibiotic use for the following common indications? (Select all that apply.)	☐ CAP<br>☐ UTI<br>☐ SSTI<br>☐ C. difficile infections<br>☐ Surgical prophylactic antibiotics<br>☐ Other ______
Does your hospital ASP evaluate adherence to at least one of the guidelines selected above?	◯ Yes<br>◯ No

Which one(s) are evaluated? (Select all that apply.)	☐ CAP<br>☐ UTI<br>☐ SSTI<br>☐ C. difficile infections<br>☐ Surgical prophylactic antibiotics<br>☐ Other ______

Reporting

Does your ASP share antibiotic use data with the following? (Select all that apply.)	☐ Hospital administration<br>☐ Front-line clinicians<br>☐ Pharmacy and therapeutics (P&T) committee<br>☐ ASP committee<br>☐ Other ______<br>☐ None of the above
Does your hospital/microbiology lab develop an annual antibiogram?	◯ Yes<br>◯ No
Does your hospital/microbiology lab develop a separate pediatric antibiogram?	◯ Yes, we make a separate antibiogram<br>◯ No, we only have a shared adult/pediatric antibiogram<br>◯ N/A, our ASP is a separate pediatric hospital with only one antibiogram

Education

Which of the following educational activities does your ASP perform? (Select all that apply.)	☐ Annual education to all prescribers (e.g., required online module)<br>☐ Lectures to trainees<br>☐ Grand Rounds<br>☐ Education targeting patients and families<br>☐ Other ______<br>☐ None of the above
Does your ASP offer rotations to any of the following trainees? (Select all that apply.)	☐ Medical Students<br>☐ Medical Residents<br>☐ Medical Fellows<br>☐ Pharmacy Students<br>☐ Pharmacy Residents<br>☐ Other ______<br>☐ None
Does your hospital/ASP provide education to prescribers as part of the prospective audit and feedback process?	◯ Yes<br>◯ No

Survey, Part II

Provider DEI

Please complete the survey below.

Thank you!

Please only complete this form for yourself. Each member of your ASP is being asked to answer these questions for themselves.

Information provided below will only be reported in aggregate at the national level. Participation in the survey, or answering any individual question, is strictly voluntary.

Please select your position:	◯ Physician<br>◯ Pharmacist<br>◯ Registered Nurse<br>◯ Nurse Practitioner<br>◯ Data Analyst<br>◯ Infection Preventionist<br>◯ Other ______
Which of the following pharmacy training have you completed?	☐ Pediatric ID<br>☐ Adult ID<br>☐ Pediatric non-ID<br>☐ Other ______
Have you completed a formal ASP training (e.g., certificate program)?	◯ Yes<br>◯ No
What hospital do you work for?	______________________________
Do you participate in inpatient or outpatient ASP?	◯ Inpatient<br>◯ Outpatient<br>◯ Both
What is your race? (Select all that apply.)	☐ American Indian or Alaska Native<br>☐ Asian<br>☐ Black or African American<br>☐ Native Hawaiian and Other Pacific Islander<br>☐ White<br>☐ Some other race ______<br>☐ Prefer not to answer
Do any of the following also apply to you? (Select all that apply.)	☐ Japanese<br>☐ Filipino<br>☐ Chinese<br>☐ Korean<br>☐ Other Asian ______

Do any of the following also apply to you? (Select all that apply.)	☐ Native Hawaiian<br>☐ Pacific Islander<br>☐ Samoan<br>☐ Tongan<br>☐ Maori<br>☐ Fijian<br>☐ Chamarro<br>☐ Cuukese<br>☐ Kosraen<br>☐ Mashallese<br>☐ Palauan<br>☐ Pohnpeian<br>☐ Yapese<br>☐ Other Pacific Islander ______
Are you of Hispanic, Latino, or Spanish origin?	◯ No, not of Hispanic, Latino, or Spanish origin<br>◯ Yes<br>◯ Prefer not to answer
Do any of the following also apply to you? (Select all that apply.)	☐ Mexican, Mexican American, Chicano<br>☐ Puerto Rican<br>☐ Cuban<br>☐ Another Hispanic, Latino, or Spanish origin ______
Which term best describes your gender identity?	◯ Woman<br>◯ Man<br>◯ Non-binary<br>◯ Transgender man/Female-to-male (FTM)<br>◯ Transgender woman/Male-to-female (MTF)<br>◯ Gender non-binary/Genderqueer/Gender nonconforming<br>◯ Agender<br>◯ Bigender<br>◯ None of these (other) ______<br>◯ Prefer not to answer
What was your assigned sex at birth?	◯ Male<br>◯ Female<br>◯ Prefer not to answer
How many years are you removed from completion of your highest level of training?	______________________
How many total years have you worked in ASP, following completion of training?	______________________
